# Neurocognitive Functioning of Adolescents with Clinical High Risk for Psychosis, other Psychiatric Symptoms, and Psychosis

**DOI:** 10.1192/j.eurpsy.2022.819

**Published:** 2022-09-01

**Authors:** M. Orlandi, M. Iorio, C. Rogantini, A. Vecchio, C. Coci, E. Casini, R. Borgatti, M. Mensi

**Affiliations:** 1 Fondazione Mondino - Istituto Neurologico Nazionale IRCCS, Child Neuropsychiatry, Pavia, Italy; 2 University of Pavia, Department Of Brain And Behavioural Sciences, Pavia, Italy

**Keywords:** schizophrénia, neurocognitive profiles, PSYCHOTIC DISORDERS, Adolescents

## Abstract

**Introduction:**

Clinical High Risk of Psychosis (CHR-P) condition and the clinical validity of at-risk criteria are still little studied in child and adolescent population.

**Objectives:**

This study aimed to discover neurocognitive profiles of adolescents with CHR-P, compared with adolescents with psychosis and youth with other psychiatric symptoms that do not meet CHR-P criteria.

**Methods:**

We divided 116 adolescents (12-18 years old) in three groups according to the semi-structured interview Comprehensive Assessment of At-Risk Mental States (CAARMS): psychosis, attenuated psychosis syndrome (APS), non-APS. Moreover, we administered Wechsler scales to assess the IQ, Wisconsin Card Sorting Test to assess abstract reasoning and flexibility, Rey-Osterrieth complex figure to assess planning and attention, and Trail Making Test to assess psychomotor speed, visual attention and task switching. We administered BVN 12-18 subtests to assess lexical denomination, verbal and nonverbal working memory, selective auditory, visual attention, phonemic and categorial fluency, reasoning and problem solving.

**Results:**

Nineteen adolescents met criteria for psychosis, 47 for APS, and 50 did not meet criteria neither for psychosis nor for APS. APS group performed better than psychosis group and similar to non-APS group in processing speed, planning, visual attention, and categorial fluency. APS did not show a significant difference from the other groups in working memory and backward digit span, showing an intermediate profile; non-APS and psychosis groups still differed significantly in these functions.

**Conclusions:**

Identifying typical neurocognitive profiles leads to more accurate diagnoses and early intervention that can lead to better patient outcomes.

**Disclosure:**

The authors declare that they do not have a significant financial interest, consultancy or other relationship with products, manufacturer(s) of products or providers of services related to this abstrac.

